# Silicon Promotes Growth of *Brassica napus* L. and Delays Leaf Senescence Induced by Nitrogen Starvation

**DOI:** 10.3389/fpls.2018.00516

**Published:** 2018-04-23

**Authors:** Cylia Haddad, Mustapha Arkoun, Franck Jamois, Adrian Schwarzenberg, Jean-Claude Yvin, Philippe Etienne, Philippe Laîné

**Affiliations:** ^1^Normandie Université, Caen, France; ^2^UMR 950 Ecophysiologie Végétale, Agronomie et Nutritions NCS, Université de Caen Normandie, Caen, France; ^3^Institut National de la Recherche Agronomique, UMR 950 Ecophysiologie Végétale, Agronomie et Nutritions NCS, Caen, France; ^4^Centre Mondial de l’Innovation, Groupe Roullier, Saint-Malo, France

**Keywords:** N privation, N uptake, *SAG12/Cab* indicator, leaf life span, photosynthetic activity

## Abstract

Silicon (Si) is the second most abundant element in soil and has several beneficial effects, especially in plants subjected to stress conditions. However, the effect of Si in preventing nitrogen (N) starvation in plants is poorly documented. The aim of this work was to study the effect of a short Si supply duration (7 days) on growth, N uptake, photosynthetic activity, and leaf senescence progression in rapeseed subjected (or not) to N starvation. Our results showed that after 1 week of Si supply, Si improves biomass and increases N uptake and root expression of a nitrate transporter gene. After 12 days of N starvation, compared to -Si plants, mature leaf from +Si plants showed a high chlorophyll content, a maintain of net photosynthetic activity, a decrease of oxidative stress markers [hydrogen peroxide (H_2_O_2_) and malondialdehyde (MDA)] and a significant delay in senescence. When N-deprived plants were resupplied with N, a greening again associated with an increase of photosynthetic activity was observed in mature leaves of plants pretreated with Si. Moreover, during the duration of N resupply, an increase of N uptake and nitrate transporter gene expression were observed in plants pretreated with Si. In conclusion, this study has shown a beneficial role of Si to alleviate damage associated with N starvation and more especially its role in delaying of leaf senescence.

## Introduction

In the lithosphere, silicon (Si) is the second most abundant element after oxygen both in terms of weight and number of atoms (Epstein, 1994). Despite this high abundance in soil, Si is never found in a free form but usually combined with other elements to form oxides compounds or silicates, which are not available for plant nutrition. Thus, only a low proportion of Si is taken up by roots in the form of uncharged silicic acid [Si(OH)_4_], which is present in the soil solution at concentrations ranging from 0.1 to 0.6 mM (Epstein, 1994). This Si root uptake involves specific Low Silicon 1 (LSi1) channel identified for the first time in rice by Ma and Yamaji (2006). Si can be then translocated to the shoots thanks to two other Si transporters, i.e., LSi2 [which allows Si efflux outside the Casparian strip in the roots (Ma et al., 2007)] and LSi6 expressed only in leaves. Subsequently, Si is polymerized and accumulated in amorphous forms in plant tissues (SiO_2_-nH_2_0) (Bauer et al., 2011). Because of the ubiquitous presence of Si in the environment, vascular plants accumulate large ranges of Si, from 0.1 to 15% of dry weight (DW) (Epstein, 1999) and agricultural crops are usually classified into three main groups (weak, medium, and strong Si accumulators) according to their Si contents. Thus, dicotyledon species with low Si contents (around 0.1% of DW) are classified as “weak Si accumulators.” Monocotyledon crops are considered either as “intermediate accumulators” if Si content is between 1–3% of DW (as rye, oats, or wheat) or as “strong accumulators” if their Si content reach 15% of DW (as cultivated rice) (Jones and Handreck, 1967; Epstein, 1999; Broadley et al., 2011).

Although Si is not an essential nutrient for most plants, it is considered to have a beneficial effect on improving resistance against biotic and abiotic stresses and agricultural crop quality and yield (Epstein, 1994, 1999; Korndorfer and Lepsch, 2001). For example, in some plants such rice, *Arabidopsis* and sugarcane, Si confers a protective role and improves pest and pathogen resistance (Ishiguro, 2001; Meyer and Keeping, 2001; Fauteux et al., 2006). Some studies have also shown that Si enhances plant tolerance against heavy metals, drought, salinity and nutrient deficiencies (Epstein, 1999; Chen et al., 2016). These beneficial effects were usually attributed to the mechanical role of Si (for example by deposition of opal or phytoliths), which acts by reinforcing cell walls and thus increases resistance to many biotic and abiotic stresses (Fauteux et al., 2005). Moreover, some authors suggest that the role of Si is more complex and that soluble Si can also act as a signal to modulate metabolic pathways (Samuels et al., 1991; Fawe et al., 2001; Fauteux et al., 2005). Indeed, some studies have shown that Si treatment of plants alleviates stress response genes (Fauteux et al., 2006) and induces an accumulation of enzymes involved in photosynthesis and detoxification of reactive oxygen species (Savant et al., 1997; Schmidt et al., 1999) and also phytohormone synthesis (Rodrigues et al., 2015; Van Bockhaven et al., 2015). In cucumber, Adatia and Besford (1986) have also observed that Si treatment increases leaf area, leaf erectness chlorophyll and RuBisCo contents. More recently, Markovich et al. (2017) have shown in detached *Arabidopsis* leaves overexpressing a rice Si channel (LSi1) that Si treatment promotes a delay of leaf senescence induced by darkness.

Taken together, all these studies suggest that Si could increase the leaf life span of plants, particularly when grown under stress conditions. This hypothesis may be of great interest for agricultural crops, such as *Brassica napus* L., characterized by low nitrogen (N) efficiency especially due to its inefficient endogenous leaf N remobilization associated with senescence (Avice and Etienne, 2014). Thus, it has been observed that senescent leaves fall with high N content (2–2.5% of dry weight; Rossato et al., 2001; Malagoli et al., 2005b). As demonstrated by Malagoli et al. (2005a) using a mechanistic model, an extension of the leaf life span could improve N transfer from vegetative to reproductive tissues and consequently allow an increase in yield (around +15%) or N seed content. To our knowledge, apart from works focused on the effect of Si nutrition in alleviating salinity or heavy metal stress (Hashemi et al., 2010; Farshidi et al., 2012; Hasanuzzaman et al., 2017), no studies have explored the effect of Si on growth and leaf senescence in *Brassica napus* grown under N starvation.

The aim of this study is to evaluate the effects of a short duration of Si treatment (7 days) on the growth, N uptake and photosynthetic activity of *Brassica napus* L. subjected (or not) to N starvation and then resupply with N. In addition, during this experiment, the Si effect on (i) root and mature leaf cytokinin contents, (ii) oxidative stress markers in mature leaf, and (iii) temporal leaf senescence progression was closely monitored using physiological and molecular senescence indicators.

## Materials and Methods

### Plant Growth Conditions and Experimental Design

**FIGURE 1:**
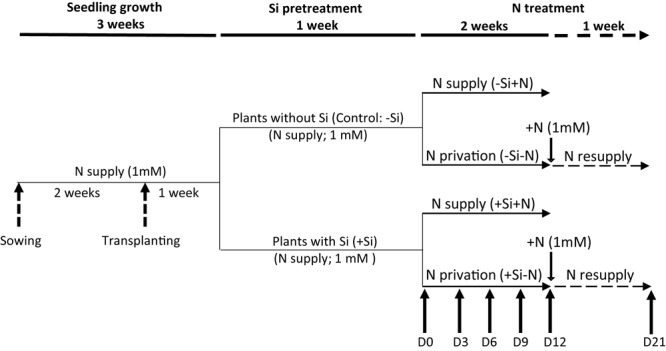
Experimental design used to study the effect of silicon pretreatment on growth parameters of *Brassica napus* L. cultivated with or without nitrogen for 12 days and then resupplied with N (from Day 12 to 21). After a period of pretreatment for 1 week with (+Si: 1.7 mM Na_2_SiO_3_) or without silicon (control: –Si), plants were separated into two groups: the first was supplied with 1 mM of NH_4_NO_3_ (–Si+N and +Si+N) and the second was N deprived (–Si–N and +Si–N). Day 0 (D0) corresponds to the end of the Si pretreatment and the beginning of the N treatment. After 12 days (D12), N-deprived plants were resupplied with 1 mM of NH_4_NO_3_ until 21 days after the end of the Si pretreatment (D21). Plants were harvested after 0, 3, 6, 9, 12, and 21 days for N-deprived plants (dashed arrows).

In a greenhouse, seeds of *Brassica napus* L. var. “Citizzen” were germinated on perlite over deionized water for 4 days in the dark. Then seedlings were transferred to natural light conditions and supplied with nutrient solution for 10 days containing: NH_4_NO_3_ (1 mM), K_2_SO_4_ (1 mM), KH_2_PO_4_ (0.4 mM), K_2_HPO_4_ (0.15 mM), CaCl_2_ (3 mM), MgSO_4_ (0.5 mM), EDTA-2NaFe (0.2 mM), H_3_BO_3_ (14 μM), MnSO_4_ (5 μM), ZnSO_4_ (3 μM), CuSO_4_ (0.7 μM), (NH_4_)_6_Mo_7_O_24_ (0.7 μM), CoCl_2_ (0.1 μM), and NiCl_2_ (1 μM). Just after first leaf emergence (i.e., after 2 weeks), seedlings were transferred for 1 week into a plastic tank (20 L) containing the nutrient solution described above. Natural light was supplied by high pressure sodium lamps (Philips, MASTER GreenPower T400W) with a PAR (Photosynthetically Active Radiations) of 450 μmol photons⋅m^-2^⋅s^-1^ at canopy height. At the emergence of the fourth leaf, silicon (Si) pretreatment was applied for 1 week (**Figure 1**). For this, plants were separated into two groups: the first (+Si) was supplied with the nutrient solution described above with the addition of 1.7 mM silicon (Si as sodium metasilicate: Na_2_SiO_3_⋅9H_2_O) and the second (-Si; Control) with the nutrient solution plus NaCl (3.4 mM) to compensate the sodium supplied by sodium metasilicate in the +Si treatment. Nutrient solutions were aerated and renewed every 3 days and their pH was adjusted daily to 5.6 with HCl (or NaOH). After the Si pretreatment period (Day 0), plants were again divided into two groups: one half of each of the +Si and –Si plants was supplied with 1mM of NH_4_NO_3_ (-Si+N and +Si+N) and the other halves were N deprived (-Si-N and +Si-N). All plants were grown in these N conditions for 12 days (D12). Then, N-deprived plants (-Si-N or +Si-N) were resupplied with 1 mM NH_4_NO_3_ for 9 days (D21) to study the effect of Si pretreatment on their capacity to take up newly supplied N (**Figure 1**). Throughout the experiment, plants were grown with a thermoperiod of 20/17°C day/night and a photoperiod of 16 h.

Plants were harvested at 0, 3, 6, 9, and 12 days and at 21 days for N-deprived plants, which were resupplied with N (**Figure 1**). At each harvest, shoots and roots were separated, and then from shoot, mature leaf corresponding to leaf with maximal area and biomass was separated (**Supplementary Figure S1**). Different tissues were frozen in liquid N and stored at -80°C for further analysis. An aliquot of each tissue was dried in an oven (60°C) for DW determination.

### Chlorophyll Contents and Net Photosynthetic Activity of the Mature Leaf

According to Ruiz-Espinoza et al. (2010), a SPAD-502 chlorophyll meter (Soil Plant Analysis Development; Minolta, model SPAD-502) was used as a relevant non-destructive method to estimate leaf chlorophyll content. The measurements of SPAD values were performed every day in mature leaves selected at the beginning of N starvation (D0). After 2 hours of illumination, net photosynthetic activity was monitored on the same leaf using a LI-6400 (LI-COR, Lincoln, NE, United States) at 500 μmol⋅m^-2^⋅s^-1^ photosynthetic photon flux (PPF) provided by a LED light.

### Total Nitrogen and Silicon Analysis

An aliquot of dry plant tissue was crushed to powder and placed in a capsule for isotopic analysis to analyze between 60 and 80 μg N. The total N was determined with a continuous flow isotope mass spectrometer (Isoprime, GV Instruments, Manchester, United Kingdom) linked to a C/N/S analyser (EA3000, EuroVector, Milan, Italy).

Silicon contents in plants tissues were determined at day 0 using a colorimetric method previously described by Dai et al. (2005). Briefly, 100 mg of dried and crushed plants tissues were added to 3 ml of NaOH (50%) and autoclaved at 121°C for 20 min. After adjustment of the volume to 10 ml with ddH_2_O, 160 μl of sample solution were transferred into a 2 ml tube containing 1.2 ml of acetic acid (20%). Then 400 μl of ammonium molybdate solution (43.7 mM, pH 7) were added and mixed thoroughly. After 5 min, 200 μl of tartaric acid (20%) and 40 μl of reducing solution were added (made by mixing solution A: 2 g of Na_2_SO_3_ and 0.4 g of 1-amino-2-naphthol-4-sulfonic acid in 25 mL of dH_2_O and solution B: 25 g of NaHSO_3_ in 200 ml of ddH_2_O and adjusted to 250 ml with ddH_2_O). After 30 min, the absorbance was measured at a wavelength of 650 nm and the silicon content was determined by referring to a standard curve prepared from a silicon solution standard at 1000 SiO_2_ mg⋅L^-1^. All reagents were stored in plastic bottles.

### RNA Extraction, Reverse Transcription, Q-PCR and Semi Quantitative PCR Analysis

Total RNAs were extracted from 200 mg of mature leaves or roots. Samples were ground to a powder with a pestle in a mortar containing liquid nitrogen. The resulting powder was mixed with 750 μl extraction buffer (0.1 M TRIS, 0.1 M LiCl, 1 mM EDTA and SDS10% (W/V), and pH8) and 750 mM of hot phenol (80°C, pH4), and the mixture was vortexed for 40 s. After the addition of chloroform/isoamyl alcohol (24:1), the homogenate was centrifuged at 15000 *g* (5 min at 4°C). The supernatant was transferred into 750 μl of LiCl solution (4 M) and incubated overnight at 4°C. After centrifugation (15000 *g* for 30 min at 4°C), the pellet was suspended in 100 μl of sterile water. Purification of RNAs including a step of DNA digestion by DNAse treatment was performed using RNeasy mini kit according to the manufacturer’s protocol (Qiagen, Courtaboeuf, France). Quantification of total RNAs was performed by spectrophotometry at 260 nm (BioPhotometer, Eppendorf, Le Pecq, France) before Reverse Transcription (RT) analysis and the quality of RNAs (**Supplementary Figure S3**) is monitored by separation of 1 μg of total RNAs on agarose gel (1% w/v) containing ethidium bromide (0.5 μg/ml). For reverse transcription, 1 μg of total RNAs was converted to cDNA with an iScript cDNA synthesis kit using the manufacturer’s protocol (Bio-Rad) before quantitative (Q-PCR) or semi quantitative PCR analysis.

For Q-PCR amplification the following primers were selected: *EF1* (Forward: 5′-TTTCGAGGGTGACAACATGA-3′; Reverse: 5′-CCGTTCCAATACCACCAATC-3′) and *18S* (Forward: 5′-CGGATAACCGTAGTAATTCTAG-3′; Reverse: 5′-GTACTCATTCCAATTACCAGAC-3′) as housekeeping genes. *BnaNRT1.1* (Forward: 5′-ATGGTAACCGAAGTGCCTTG-3′; Reverse: 3′-TGATTCCAGCTGTTGAAGC-5′), *BnaNRT2.1* (Forward: 5′-TGGTGGAATAGGCGGCTCGAGTTG-3′; Reverse: 5′-GTATACGTTTTGGGTCATTGCCAT-3′), and *BnaAMT1.1* (Forward: 5′-GTCCTTGACGCTGCAGCCGGTG-3′; Reverse: 5′-CGGGCTGGCCCATCCATCAAC-3′) as target genes. The Q-PCR reactions were performed with 4 μL of 200X diluted cDNA, 500 nM of the primers and 1X SYBER Green PCR Master Mix (Bio-Rad) in a total volume of 15 μL in a ChromoFour System (Bio-Rad). For each pair of primers, a threshold value and PCR efficiency were determined using a cDNA preparation diluted >10-fold. For both pairs of primers, PCR efficiency was ≈100%. The specificity of PCR amplification was examined by monitoring the presence of the single peak in the melting curves after Q-PCRs and by sequencing the Q-PCR product to confirm that the correct amplicons were produced from each pair of primers. The relative expression of the target gene in each simple was compared to the control sample and was determined with the delta-delta Ct method using the following equation:

$$\begin{array}{c} \mathrm{Relative}\;\mathrm{expression}=2^{-[\Delta \mathrm{Ct}\;\mathrm{sample}-\Delta \mathrm{Ct}\;\mathrm{control}]} \end{array}$$

With With ΔCt = Ct_target gene_ – Ct_housekeeping genes_

Where the Ct of housekeeping genes is the geometric mean between the Ct of two housekeeping genes.

Relative expression of the different nitrogen transporter genes in the control sample was made equal to one and the relative expression of the other treatments was then compared with the control (Livak and Schmittgen, 2001).

According to Gombert et al. (2006), analysis of *BnaSAG12* and *Cab* gene expression was performed using semi quantitative PCR. Reactions were performed using specific primers for the *Brassica napus Cab* gene *LHCII type I* (Forward: 5′-GGCAGCCCATGGTACGGATC-3′; reverse: 5′-CCTCCTTCGCTGAAGATCTGT-3′), *BnaSAG12* (Forward: 5′-GGCAGTGGCACACCAMCCGGTTAG-3′; reverse: 5′-AGAAGCMTTCATGGCAAGACCAC-3′) as target genes and *EF1-α* (Forward: 5′-TTTCGAGGGTGACAACATGA-3′; reverse: 5′-CCGTTCCAATACCACCAATC-3′) as an internal control gene (Nicot et al., 2005). PCRs were performed with Qbiogene Taq polymerase (MP Biomedicals, Illkirch, France) on a thermocycler (Applied Biosystems, Courtaboeuf, France) according to the manufacturer’s protocol. The amplification program was as follows: 1 cycle at 95°C for 5 min, 20 for *Cab* and *BnaSAG12* and 26 for *EF1-α* cycles including a denaturing step at 95°C for 30 s, a primer’s hybridization step at 58°C for 45 s and an amplification step at 72°C for 1 min. Each PCR reaction was finished with one cycle at 72°C for 10 min. Four single 267, 220, 161, and 164 bp cDNAs were amplified for the *BnaSAG12, Cab*, and *EF1-α* genes, respectively, and the identity of each fragment was checked by sequencing (Biofidal, Vaulx-en-Velin, France). RT-PCR products were separated by electrophoresis via agarose gels (1%) containing ethidium bromide (0.5 μg/ml). These agarose gels were scanned under UV light with a Gel Doc^TM^ EZ scanner (Bio-Rad, Marnes-la-Coquette, France) and the transcript levels were quantified with ImageLabTM software (Bio-Rad, Marnes-la-Coquette, France) after normalization with the *EF1-α* gene. The data related to the *BnaSAG12* and *Cab* transcript levels were expressed as a percentage of the maximum of both transcripts observed in mature leaves (the maximum level of transcripts was observed at day 21 for *BnaSAG12* and day 0 for *Cab*). The date of entry into senescence of mature leaves was determined according to the method of Gombert et al. (2006); the theoretical time (Th.T.) of senescence for a given leaf rank is determined using the time course of expression of *BnaSAG12* (up-regulated during senescence) and *Cab* (down-regulated during senescence). The intersection point corresponding to the concomitant up-regulation of *BnaSAG12* and down regulation *Cab* genes was considered as the time of onset of foliar senescence.

### Cytokinin Analysis by UHPLC-MS/MS

To 10 mg of frozen grounded leaves and roots, 1 mL of a solution at -20°C of 70% MeOH/29% H_2_O/ 1.0% formic acid containing isotopically labeled internal standards at 0.2 ng/mL ([^2^H_3_]-Dihydrozeatin, [^2^H_3_]-Dihydrozeatin riboside, [^2^H_6_]N^6^-Isopentenyladenine, [^2^H_6_]N^6^-Isopentenyladenosine, [^2^H_5_]-*trans*-zeatin) was added. The mix was stirred at room temperature for 30 min then centrifuged (16000 rpm; 4°C). The supernatant was evaporated to dryness under nitrogen using a Turbovap LV system (Biotage, Sweden). Dry extracts were dissolved with 2 mL of a 2% formic acid solution. The extracts were purified using a solid phase extraction (SPE) Evolute express CX 3 mL-60 mg (Biotage, United Kingdom). The extracts were added in two steps to improve cytokinins retention, then the samples were washed with 1 mL of 2% formic acid / 98% H_2_O solution. A second wash was performed with 1 mL of MeOH. The elution of the cytokinins was done with 2 mL of a 95% MeOH/5% NH_4_OH solution in two steps. The eluates were evaporated to dryness and dissolved in 100 μL of H_2_O containing 0.1% of formic acid before analysis by using UHPLC-MS/MS system.

The separation and detection were achieved using a Nexera X2 UHPLC system (Shimadzu, Japan) coupled to a QTrap 6500+ mass spectrometer (Sciex, Canada) equipped with an IonDrive^TM^ turbo V electrospray (ESI) source. 2 μl of cytokinins extract was separated into a Kinetex Evo C18 core-shell column (100 mm × 2.1 mm, 2.6 μm, Phenomenex, United States) at a flow rate of 0.7 mL/min, and the column oven maintained at 40°C. The mobile phases were composed of solvent A Milli-Q water (18 MΩ, Millipore, United States) containing 0.1% formic acid (LCMS grade, Fluka analytics, Germany), and solvent B acetonitrile LCMS grade (Fisher Optima, United Kingdom) containing 0.1% formic acid. The gradient elution started with 2% B, 0.0–3.0 min 20% B, 3.0–4.0 min 25% B, 4.0–4.5 min 100% B, 4.5–6.0 min 100% B, 6.0–6.5 min 2%, and 6.5–8.5 min 2% B. The capillary voltage was set to 5kV producing mainly [M+H]^+^ ions. All quantitative data was processed using MultiQuant software V 3.0.2 (Sciex, Canada).

### Determination of Hydrogen Peroxide (H_2_O_2_) and Malondialdehyde (MDA) Contents

Hydrogen peroxide (H_2_O_2_) was determined according to the method previously described by Yu et al. (2003). H_2_O_2_ was extracted from 200 mg of fresh mature leaf homogenized with 3 ml of 50 mM potassium-phosphate buffer (pH 6.5) at 4°C. After centrifugation, (11500 × *g*; 15 min), the supernatant (3 ml) was mixed with 1 ml of 0.1% TiCl_4_ in 20% sulfuric acid (H_2_SO_4_; v/v). The mixture was then centrifuged (11500 × *g*; for 15 min) and the supernatant was used for measurement of absorbance at 410 nm. H_2_O_2_ content was calculated using 𝜀= 0.28 μM^-1^cm^-1^ as the molar extinction coefficient and expressed as μmol g^-1^ FW.

Lipid peroxidation was estimated from malondialdehyde (MDA), a decomposition product of the peroxidized polyunsaturated fatty acid component of the membrane lipid. The MDA content in mature leaf at Day 12 (-Si-N and +Si-N) was determined based on thiobarbituric acid-reacting substances (TBARS) as previously described in Hasanuzzaman et al. (2011). Briefly, fresh leaf tissue (0.2 g) were homogenized with 4 ml of 5% trichloroacetic acid (TCA) and centrifuged at 5000 × *g* during 30 min. A total of 1 mL of the supernatant was mixed with 4 mL of 0.5% TBA made in 20% TCA, incubated at 95°C for 30 min, then quickly cooled on ice, before measurement of absorbance at 532 and 600 nm. Then, the MDA content was calculated based on the difference in absorbance (A_532_–A_600_) and is expressed as nmol g^-1^ FW. For this, 𝜀= 155 mM^-1^ cm^-1^ was used as the molar extinction coefficient.

### Statistical Analysis

The experiment was performed with three replicates except for measurements at day 0, which were performed with four replicates due to the weak biomass of the plants. The resulting variations in data are expressed as the mean ± standard error (SE) for *n* = 3 (or *n* = 4 at D0). The results were subjected to statistical analysis in R software. Data were analyzed using analysis of variance (ANOVA) after verifying compliance of normality of the data and homogeneity of variance using the Shapiro–Wilk and Bartlett tests, respectively. Mean values were compared using Tukey’s test.

## Results

### Plant Growth, Si and N Uptakes and Root Cytokinin Content in *Brassica napus* Treated With Silicon (Day 0)

**FIGURE 2:**
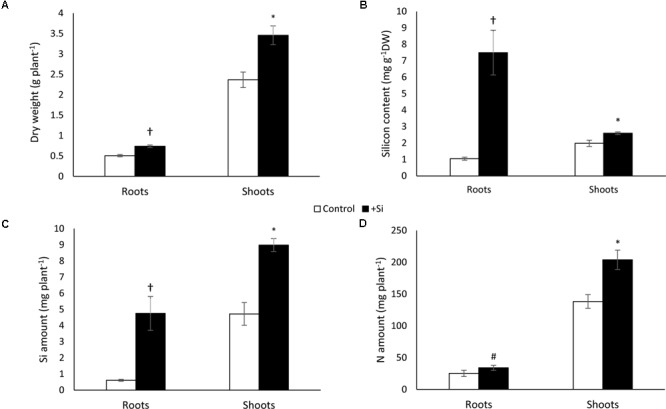
Dry weight **(A)**, total silicon amount **(B)**, silicon content **(C)** and total nitrogen amounts **(D)** in shoots and roots of *Brassica napus* L. at the end of the Si pretreatment (Day 0). This harvest time (Day 0) corresponds to the end of the pretreatment period of plants with (+Si) or without Si (–Si; Control). Data are means ± SE (for *n* = 4). ^∗^ and ^†^ indicate significant differences between –Si (control) and +Si plants with *p* < 0.05 and *p* < 0.001, respectively.

The biomass of whole plants treated with silicon (+Si) was significantly higher than control plants (-Si) and rose 4.2 mg plant^-1^ versus 2.9 mg plant^-1^, respectively. Both root and shoot biomasses were significantly higher in +Si plants and were about 0.8 and 3.5 mg, and 0.5 and 2.4 mg plant^-1^ for +Si and -Si plants, respectively (**Figure 2A**). The Si content in +Si whole plants was also significantly higher than in -Si whole plants. Consequently, the total amount of Si was 2.6-fold higher in +Si plants than control (13.73 versus 5.33 mg plant^-1^) (**Figure 2B**). Moreover, in +Si plants, Si content was significantly higher in root than in shoot content (0.75% versus 0.26%) (**Figure 2C**).

**FIGURE 3:**
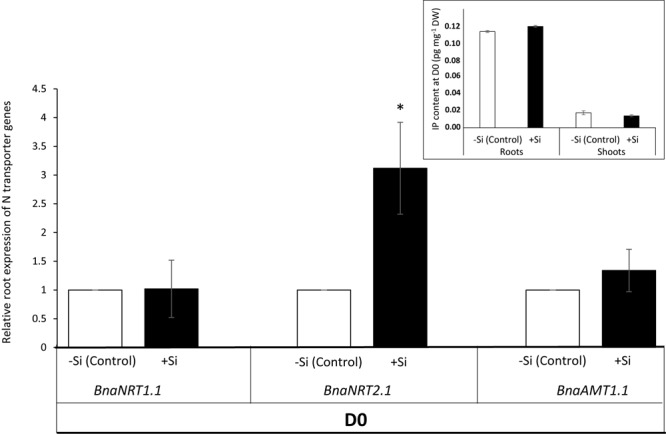
Relative root expression of the *BnaNRT1.1, BnaNRT2.1*, and *BnaAMT1.1* genes in *Brassica napus* L. at the end of the Si pretreatment period (D0). This harvest time (Day 0) corresponds to the end of the pretreatment period of plants with (+Si) or without Si (–Si; Control). Insert represents active cytokinin (Isopentenyladenine: IP) contents in roots. Detail of cytokinin contents are available in **Supplementary Table S1**. Data are means ± SE (for *n* = 4). ^∗^ indicates significant differences between –Si (control) and +Si plants with *p* < 0.05.

In +Si plants, the total N amount was significantly increased in shoot (1.50-fold) and roots (1.35-fold) compared to -Si plants (**Figure 2D**). At the same time, in +Si plants the relative expression level of *BnaNRT2.1* (encoding high affinity nitrate transporter) in the roots increased significantly (3-fold) compared to -Si plants, while the expression level of the *BnaNRT1.1* and *BnaAMT1.1* genes (encoding low affinity nitrate and ammonium transporters, respectively) remained the same (**Figure 3**). Moreover, except a decrease of root *trans*-zeatin (t-Z) content observed in plant treated with Si, all other cytokinin contents at D0 (and especially N_6_-Isopentenyladenine (IP) considered as one of the main active forms) studied in roots and shoots remained the same level in -Si and +Si plants (**Figure 3** and **Supplementary Table S1**).

**FIGURE 4:**
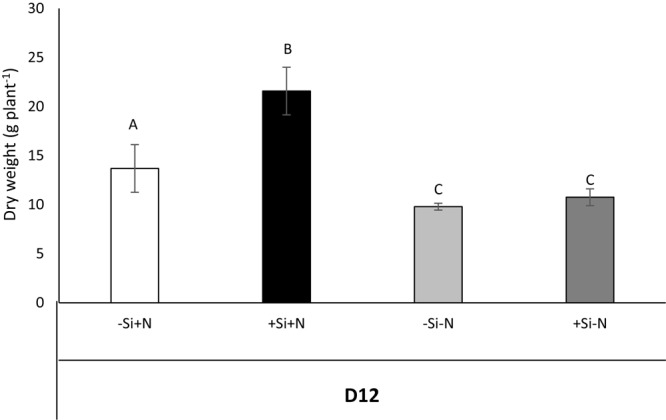
Effect of Si pretreatment on total dry weights of *Brassica napus* L. supplied or not supplied with N. During the 12 days after the Si pretreatment, control (–Si) and Si pretreated (+Si) plants were grown with (–Si+N, +Si+N) or without N (–Si–N, +Si–N). Data are means ± SE (for *n* = 3). Different capitals indicate that the means of total plant dry weights are significantly different at *p* < 0.05.

After this period of Si pretreatment, -Si and +Si plants were transferred for 12 days onto a solution deprived of Si and containing (+N) or lacking (-N) N (**Figure 4**). In +N conditions, the total biomass of plants previously treated with Si (+Si+N) was significantly higher than in plants not supplied with Si pretreatment (-Si+N). Indeed, the biomass of +Si+N plants reached 21.59 g plant^-1^ versus only 13.69 g plant^-1^ for -Si+N plants. Under -N conditions, the total biomasses of +Si and -Si plants were significantly lower than the total biomasses of plants grown under +N conditions. Moreover, whatever the Si pretreatment (+Si or -Si), the total plant biomasses (about 10 g.plant^-1^) were not significantly different (**Figure 4**).

### Effect of Si Pretreatment on the Evolution of Physiological Indicators of Leaf Senescence

**FIGURE 5:**
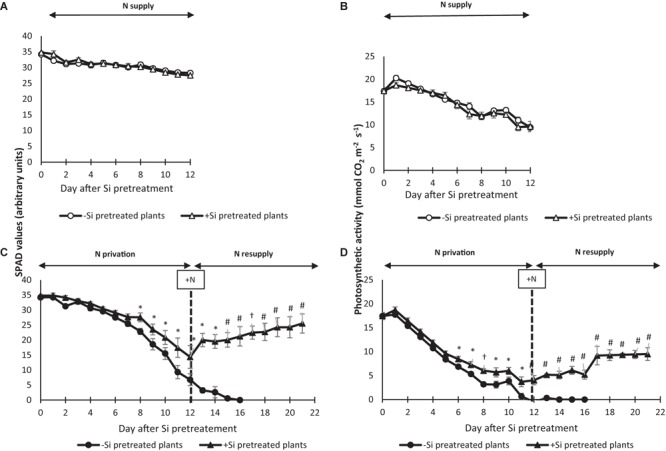
Change in SPAD values and net photosynthetic activity of mature leaf from *Brassica napus* L. Plants were pretreated with (+Si) or without (–Si) and supplied with (N supply; **A,B**) or without nitrogen (N starvation; **C,D**) for 12 days. After this time, N-deprived plants were resupplied with N (N resupply; **C,D**) for 9 days (D21). Data are means ± SE (for *n* = 3). ^∗^, #, and ^†^ indicate significant differences between –Si (control) and +Si plants with *p* < 0.05, *p* < 0.01, and *p* < 0.001, respectively.

To follow the impact of Si pretreatment on leaf senescence progression, non-destructive physiological senescence indicators (net photosynthetic activity and SPAD values) were monitored in mature leaves from plants grown with or without N for 12 days. Under +N conditions, a Si pretreatment had no effect on SPAD values or the photosynthetic activity of mature leaves compared to control (-Si) (**Figures 5A,B**). From day 0 to day 12 the SPAD values and net photosynthetic activity of mature leaves of -Si plants under N deficiency (-N) decreased from 35 to 5 SPAD units and from 18 to 0 mmol CO_2_ m^-2^ s^-1^, respectively. Compared to -Si plants, no difference in the SPAD value and net photosynthetic activity in +Si plants was observed until days 7 and 5, respectively. After these times, the values of both of these physiological parameters remained significantly higher in +Si plants than in -Si mature leaves. Thus, after 12 days of N starvation, SPAD values and net photosynthetic activity were 14.35 SPAD units (versus 6.8 in -Si plants) and 4.0 mmol CO_2_ m^-2^ s^-1^ (versus 0 in -Si plants), respectively (**Figures 5C,D**).

After this period of N starvation, -Si and +Si plants were resupplied with nitrogen from day 12 to day 21 and both physiological parameters were measured (**Figures 5C,D**). In -Si plants, net photosynthetic activity remained zero and SPAD values decreased continuously to reach 0 SPAD units at 16 days, corresponding to the time when mature leaf fall was observed. In contrast, when +Si plants were resupplied with N, increases in both physiological parameters were observed in mature leaves and reached 25.5 SPAD units and 9.5 mmol CO_2_ m^-2^ s^-1^ at day 21, respectively (**Figures 5C,D**).

### Effect of Si Pretreatment on the Evolution of Molecular Indicators Senescence and Cytokinin, Malondiadehyde and Hydrogen Peroxide Contents in Mature Leaf

**FIGURE 6:**
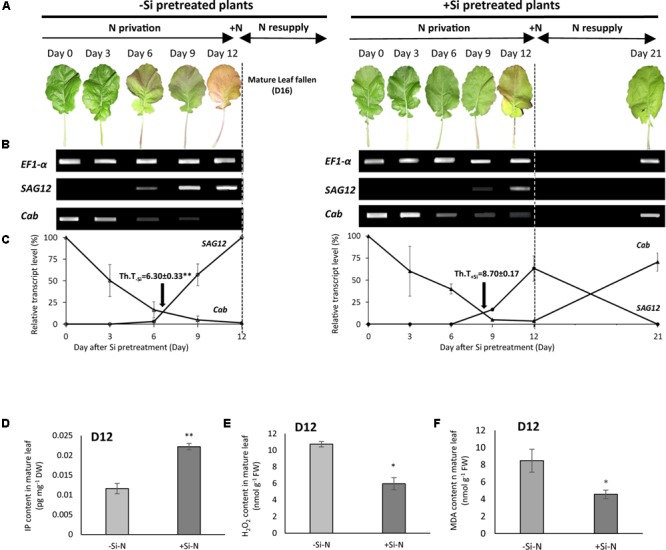
Images, changes in *SAG12/Cab* molecular senescence indicators and active cytokinin, hydrogen peroxide, and malondialdehyde contents in mature leaf from N-deprived *Brassica napus* pretreated or not with Si. **(A)** Images of mature leaf from plants pretreated without (–Si) or with Si (+Si) cultivated during 12 days without N (–N; from D0 to Day 12) and resupply with N (+N; from Day 12). **(B)** *BnaSAG12* and *Cab* gene expressions monitored by RT-PCR in mature leaves harvested after 0, 3, 6, 9, and 12 days of N starvation and 9 days after N resupply (day 21). *EF1-α* was used as a cDNA synthesis and amplification control. **(C)** Quantification of *BnaSAG12* and *Cab* transcripts using ImageLabTM software after normalization with the *EF1-α* gene. Arrows indicate the theoretical time (Th.T.) of the onset of senescence of mature leaf determined as previously described by Gombert et al. (2006). **(D)** Active cytokinin (N_6_-Isopentenyladenine: IP) content in mature leaf at 12 from plants pretreated without (–Si) or with (–Si) Si and cultivated without N (–N) during 12 days (From D0 to D12). **(E)** Hydrogen peroxide (H_2_O_2_) content in mature leaf at 12 from plants pretreated without (–Si) or with (–Si) Si and cultivated without N (–N) during 12 days (From D0 to D12). **(F)** Malondialdehyde (MDA) content in mature leaf at 12 from plants pretreated without (–Si) or with (–Si) Si and cultivated without N (–N) during 12 days (From D0 to D12). Data are means ± SE (for *n* = 3). ^∗^ and ^∗∗^ indicate significant differences between –Si and +Si plants with *p* < 0.01 and *p* < 0.05, respectively.

Considering the evolution of physiological indicators of senescence previously studied (**Figure 5**), a relevant molecular indicator of *Brassica napus* leaf senescence (*BnaSAG12/Cab*) was used to characterize the senescence progression in -Si and +Si mature leaves from plants cultivated under N starvation (from D0 to D12) and after N resupply (from D12; **Figure 6**).

The phenotypic evolution of mature leaves from -Si-N plants (**Figure 6A**) began with yellowing from day 6, and increased daily until day 9 when the leaf was fully yellow. In +Si-N plants, mature leaf yellowing only started between 9 and 12 days of N starvation.

In *Brassica napus*, previous studies have demonstrated that the onset of leaf senescence is characterized by a concomitant down and up-regulation of *Cab* and *BnaSAG12* gene expression, respectively (Gombert et al., 2006). Considering this, time course of *Cab* and *BnaSAG12* genes expression was estimated in mature leaves from N-deprived plants. The results indicated a concomitant down- and up-regulation of *Cab* and *BnaSAG12* from 6 days in -Si-N plants, respectively. In +Si-N plants the same expression pattern of *Cab* and *BnaSAG12* was observed only after 9 days of N starvation (**Figure 6C**). The kinetic quantification of *BnaSAG12* and *Cab* expression allowed determination of the Th.T. of the onset of mature leaf senescence, and was characterized by the intersection point between curves of the *Cab* and *BnaSAG12* transcript levels. Under -N conditions, Th.T was 6.3 versus 8.7 days in mature leaves of -Si and +Si plants, respectively (**Figure 6C**). All these results indicated that Si pretreatment is associated with a delay in leaf senescence (+2.4 days) in plants grown under N starvation. Surprisingly, when N deprived plants were resupplied with N (from D12), a down regulation of *BnaSAG12* and an up-regulation *Cab* gene expression were observed at D21 in mature leaf from +Si plants while the same leaf was fallen at D16 in -Si plants (**Figures 6B,C**). This data indicating a reversion of senescence process only in mature leaf of +Si plants is in agreement with the greening again but also with the photosynthetic activity recovery and the increase of SPAD of this leaf previously observed (**Figure 5**).

At the end of the N starvation period cytokinin contents (and especially IP content) were determined in +Si and -Si mature leaf from N-deprived plants, (**Figure 6D** and **Supplementary Table S1**). At D12 (corresponding to the end of N starvation), IP contents is significantly higher in mature leaf of +Si-N plants (1.9-fold; **Figure 6D**) than in mature leaf of -Si-N while other cytokinin contents [i.e., *trans*-zeatin and N_6_-Isopentenyladenosine (IPAdo)] remain at the level whatever Si treatment (**Supplementary Table S1**). Moreover, at Day 12, the oxidative stress markers, i.e., H*_2_*O_2_ and MDA were significantly lower in +Si-N than in -Si-N mature leaf. Thus, in mature leaf of +Si-N plants, H*_2_*O_2_ and MDA contents were 5.9 μmol g^-1^ FW (versus 10.7 in -Si-N) and 4.6 nmol g^-1^ FW (versus 8.5 in -Si-N), respectively.

### N Uptake and N Transporter Gene Expression After the N Resupply Period in N-Deprived Plants Previously Pretreated or Not Treated With Si

**FIGURE 7:**
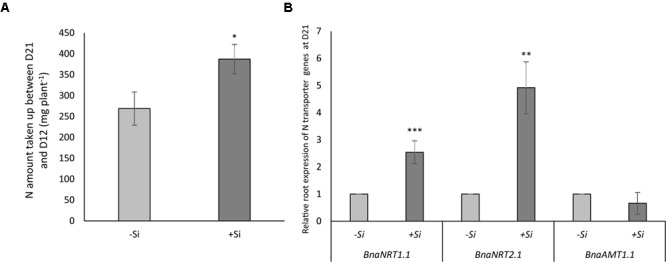
Amount of nitrogen taken up by plants between 12 (D12) and 21 days (D21; **A**), and the relative root expression of the *BnaNRT1.1, BnaNRT2.1*, and *BnaAMT1.1* genes in *Brassica napus* **(B)**. Plants were pretreated with (+Si) or without (Control: –Si) silicon after N starvation for 12 days (D12) and 9 days after N resupply (D21). Data are means ± SE (*n* = 3). ^∗, ∗∗^ and ^∗∗∗^ indicate significant differences between –Si (Control) and +Si plants with *p* < 0.05, *p* < 0.01 and *p* < 0.001, respectively.

During the 9 days of N resupply, +Si-N plants took up more N than -Si-N plants (268 versus 388 mg plant^-1^) (**Figure 7A**). This 1.5-fold increase in N uptake by +Si-N plants was associated with root up-regulation of the nitrate transporter genes, *BnaNRT1.1* and *BnaNRT2.1.* Indeed, compared to -Si-N, the relative expression levels of *BnaNRT1.1* and *BnaNRT2.1* in +Si-N plant roots were increased by 2.5 and 4.9-fold, respectively. At the same time, no effect of Si pretreatment was observed on root expression of the ammonium transporter, *BnaAMT1.1* (**Figure 7B**).

## Discussion

The aim of this study was to evaluate the effect of a short pretreatment with silicon (1 week) on growth, N uptake, chlorophyll content, net photosynthetic activity and temporal leaf senescence progression induced by N starvation in *Brassica napus* L.

Our study showed that *Brassica napus*, which is considered a non-Si accumulator species (<10 mg g^-1^ DW; Guntzer et al., 2012), is able to take up and store Si in roots in particular (**Figures 2B,C**). This result is in agreement with previous work that has shown Si root storage in *Brassica napus* grown in hydroponic conditions with Si (Farshidi et al., 2012).

After a short period of Si treatment (7 days with 1.7 mM of Si), the growth of +Si+N plants is increased by 1.45-fold compared to -Si plants. This beneficial effect of Si on plant biomass is sustainable only if plants are supplied with nitrogen (**Figure 4**). To our knowledge, this work is the first to demonstrate a beneficial effect of Si on growth of unstressed plants. Indeed, the beneficial effect of Si on growth has been shown mainly in plant species subjected to a wide range of abiotic stresses such as drought or salinity (Eneji et al., 2008; Tuna et al., 2008; Da Cunha and Do Nascimento, 2009). This biomass increase is associated with an increase in the amount of N in + Si+N plants (**Figure 2D**) and with induction (by threefold) of expression of the gene that encodes the *BnaNRT2.1* nitrate transporter (**Figure 3**). Considering that Si may promote cytokinin biosynthesis (Markovich et al., 2017) and that cytokinin is one of factors which may modulate expression of high affinity nitrate transporters (HATS) encoded by *NRT2.1* gene (Gessler et al., 2004; Dluzniewska et al., 2006; Sakakibara et al., 2006), root cytokinin contents have been monitored in plant pretreated with or without Si (**Figure 3** and **Supplementary Table S1**). Our results show that whatever Si pretreatment (+Si or -Si), IP contents (considered as one of the main active cytokinin forms) remained at the similar level. Moreover, a decrease of *trans-*zeatin riboside contents was observed in roots of +Si plants. Some studies shown that only an increase of cytokinin levels is able to modulate (up- and down-regulation) *NRT2.1* gene expression (Collier et al., 2003; Gessler et al., 2004; Sakakibara et al., 2006). Consequently, in our study, the induction of *NRT2.1* observed in root of +Si plant can not be explained by an increase of cytokinin levels. Thus, in this study, it can be suggested that Si may act as a signal able to induce nitrate transporter gene expression in *Brassica napus* as previously shown in corn salad where an induction of a Low Affinity nitrate Transporter (LATS) by Si has been demonstrated by Gottardi et al. (2012).

Under N starvation (from Day 0 to 12), this study has shown that after days 5 and 7 the net photosynthetic activity and leaf SPAD values of +Si plants were significantly higher than -Si plants, respectively (**Figures 5C,D**). In another study performed in *Zea mays* L., we have also observed a preservation of chlorophyll content in mature leaf from -N+Si plants compared to -N-Si plants (**Supplementary Figure S2**). According to Latef and Tran (2016) which have shown that seed-priming with Si improved photosynthetic pigment contents in leaves of maize exposed to alkaline stress, our results suggest that the beneficial effect of Si on leaf senescence induced by N starvation is not specific to *Brassica napus* L. This result is confirmed by the use of specific *Brassica napus* molecular indicators of leaf senescence (*BnaSAG12*/*Cab*) that showed that the progression of leaf senescence in +Si plants was shifted by 2.6 days compared -Si plants. Moreover, at Day 12, this delay of mature leaf senescence observed in +Si-N plants is associated with a significant increase of leaf IP contents (1.9-fold) comparatively to -Si-N plants (**Figure 6D**). These results were in agreement with a recent work undertaken in detached leaves of *Arabidopsis thaliana* overexpressing a silicon root transporter of rice and showing that the decrease of chlorophyll degradation and delay of leaf dark-induced senescence in Si treated plants is associated with an induction of cytokinin biosynthesis and increase of leaf content of this phytohormone (Markovich et al., 2017). Moreover, in this study, Si pretreatment induces a significant reduction of oxidative stress markers such as H_2_O_2_ and MDA in mature leaf (**Figures 6E,F**). This result is consistent with some studies showing that Si improve the antioxidant capacity and the activity in some antioxidant enzymes in different plants species subjected to abiotic or biotic stresses (Habibi, 2016; Latef and Tran, 2016; Hasanuzzaman et al., 2017). Thus in our study the delay of leaf senescence observed in +Si-N plant could be the consequence of the increase of leaf cytokinin content and/or by a better antioxidant capacity (**Figures 6E,F**).

Surprisingly, this work has shown that when N-deprived plants were resupplied with N (days 12–21), senescing mature leaves from +Si plants recovered net photosynthetic activity (**Figure 5B**) and turned green again (**Figures 5A, 6A**), while mature leaves from -Si plants continue to senesce and fall at day 16. This result is reinforced by the down-regulation of *BnaSAG12* concomitantly with up-regulation of *Cab* observed at day 21 in mature leaves of +Si plants (**Figure 6A**), confirming a reversal of leaf senescence progression only in plants pretreated with Si. Zimmermann and Zentgraf (2005) have detailed the chronological events occurring during leaf senescence and have shown that the progression of this process is triphasic (i.e., initiation, reorganization and terminal phases) with both of the first phases considered as reversible in contrast to the last, which are irreversible and lead to the death and abscission of the leaf. In this study, silicon generates a delay in leaf senescence in +Si plants compared to -Si plants. Previous works have demonstrated that *BnaSAG12* gene encoding a cysteine protease was induced during early stage to late stage of leaf senescence (Gombert et al., 2006; Desclos-Théveniau et al., 2015). Thus, the down regulation of *BnaSAG12* in mature leaf from +Si-N plants resupplied with N suggests that leaf senescence was either in initiation or reorganization phases, i.e., in a reversible phase. In contrast, the abscission of the mature leaf from -Si-N plants resupplied with N confirms that after the duration of N starvation, leaf senescence was already engaged in the irreversible terminal phase.

Finally, during this study, it was also shown after a period of N resupply that the +Si-N plants were able to take up a greater amount of nitrogen compared to -Si-N plants (**Figure 7A**). This increase in N uptake is associated with the higher root level expression of genes encoding nitrate transporters (*BnaNRT1.1* and *BnaNRT2.1*) in +Si plants (**Figure 7B**). This long-term Si effect (compared to the effect observed during Si apply) may be the indirect consequence of the maintenance of photosynthetic activity, which allows the synthesis of sufficient carbohydrate substrates to regulate the number and/or activity of nitrate transporters (Lejay et al., 2003; Feng et al., 2011). For the *Brassica napus* crop, the benefit effect of Si supply and especially the improvement of recovery capacity after a period of abiotic stress is very interesting. Indeed, it could be suggest that Si autumnal supply could alleviate stress of plants during winter season and thus promote a better growth at the beginning of the spring and promote yield.

## Conclusion

This study is the first to demonstrate a beneficial effect of Si in preventing N starvation in *Brassica napus*. Indeed, in addition to a decrease of oxidative stress markers (H_2_O_2_ and MDA) and a delay in leaf senescence during N starvation associated with an increase of leaf cytokinin content (especially IP), Si treatment is associated with reversal of this process when N nutrition conditions are again favorable. Thus, the resulting stay green effect in the leaf may allow an increase in leaf life span and promote a better synchronism between leaf senescence and seed filling (Ray et al., 1983). Indeed, many authors (Malagoli et al., 2005a; Gregersen et al., 2013) have suggested that an increase in the leaf life span of plants with low nitrogen use efficiency (such as *Brassica napus*; Avice and Etienne, 2014) may improve the remobilization of endogenous N from leaves to seeds and increase the yields of these crops. Moreover, our study like Markovich’s suggest that Si is able to act as a cellular signal able to module directly or/and indirectly gene expression. Future experiments using large-scale molecular approaches will need to be performed to explore the metabolic pathways modulated by Si and open new investigation ways.

## Author Contributions

CH, PE, PL, MA, and J-CY designed the experiments. CH, PE, and PL conducted all the experiments, analyzed the data, and wrote the paper. AS and FJ performed the cytokinin analysis.

## Conflict of Interest Statement

The authors declare that the research was conducted in the absence of any commercial or financial relationships that could be construed as a potential conflict of interest.

## Supplementary Material

FIGURE S1 Phenotype of *Brassica napus* L. plants cultivated without (-Si+N; **A**) or with silicon (+Si+N; **B**) during 1 week (D0) and grown without N for 12 days (-Si-N and +Si-N; **C,D**). The black arrows indicate the mature leaf identified at the beginning of the N starvation (D0) and used to follow the time of course of photosynthetic activity, SPAD measurements senescence progression (*BnaSAG12*/*Cab*).

FIGURE S2 Effect of Si treatment on SPAD values of mature leaf from *Zea mays* L. Plants were pretreated with (+Si) or without (-Si) during 1 week and supplied with (+N) or without N (-N) for 21 days (D21). Data are means ± SE (for *n* = 4). ^∗^ indicates significant differences between -Si (control) and +Si plants with *p* < 0.001.

FIGURE S3 Control quality of total RNAs extracted from mature leaf of N (-N) deprived plants treated with (+Si) or without (-Si). At each time (Day 0, 3, 6, 9, 12, and 21 only for +Si-N), three biological repetitions have been performed. 1 μg of total RNAs were separated on agarose gel (1%) containing 0.5 μg/ml of ethidium bromide (0.5 μg/ml). After separation, agarose gels were scanned under UV light with a Gel Doc^TM^ EZ scanner (Bio-Rad, Marnes-la-Coquette, France) and visualized using ImageLabTM software (Bio-Rad, Marnes-la-Coquette, France).

TABLE S1 Detail of cytokinin contents [expressed in pg mg^-1^ dry weight (DW)] in roots and shoots (D0) and in mature leaf and roots (D12) of *Brassica napus* L. Plants were pretreated with (+Si) or without (-Si) silicon for 1 week and supplied with (-Si+N; +Si+N) or without (-Si-N; +Si-N) N for 12 days (from D0 to D12). Data are means ± SE (for *n* = 3). ^∗^ and ^∗∗^ indicate significant differences (in bold) between -Si and +Si plants with *p* < 0.01 and *p* < 0.001, respectively. IP, N_6_-Isopentenyladenine; t-Z, *Trans*-zeatin; IPAdo, N_6_-Isopentenyladenosine.

## References

[B1] Adatia M. H. Besford R. T. (1986). The effects of silicon on cucumber plants grown in recirculating nutrient solution. *Ann. Bot.* 58 343–351. 10.1093/oxfordjournals.aob.a087212

[B2] Avice J. C. Etienne P. (2014). Leaf senescence and nitrogen remobilization efficiency in oilseed rape (*Brassica napus* L.). *J. Exp. Bot.* 65 3813–3824. 10.1093/jxb/eru177 24790115

[B3] Bauer P. Elbaum R. Weiss I. M. (2011). Calcium and silicon mineralization in land plants: transport, structure and function. *Plant Sci.* 180 746–756. 10.1016/j.plantsci.2011.01.019 21497710

[B4] Broadley M. Brown P. Cakmak I. Feng M. J. Zed R. Zhao F. (2011). “Beneficial elements,” in *Marschner’s Mineral Nutrition of Higher Plants* ed. Marschner P. (New York, NY: Elsevier Ltd) 249–270.

[B5] Chen D. Cao B. Wang S. Deng X. Yin L. Zhang S. (2016). Silicon moderated K deficiency by improving the plant-water status in sorghum. *Sci. Rep.* 6:22882. 10.1038/srep22882 26961070 PMC4785406

[B6] Collier M. D. Fotelli M. Nahm M. Kopriva S. Rennenberg D. E. Gessler A. (2003). Regulation of nitrogen uptake by *Fagus sylvatica* on a whole plant level-interactions between cytokinins and soluble N compounds. *Plant Cell Environ.* 26 1549–1560. 10.1046/j.1365-3040.2003.01079.x

[B7] Da Cunha K. P. V. Do Nascimento C. W. A. (2009). Silicon effects on metal tolerance and structural changes in maize (*Zea mays* L.) grown on a cadmium and zinc enriched soil. *Water Air Soil Pollut.* 197 323–330. 10.1007/s11270-008-9814-9

[B8] Dai W. E. Zhang K. Q. Duan B. W. Sun C. W. Zheng K. L. Cai R. (2005). Rapid determination of silicon content in rice. *Rice Sci.* 12 145–147.

[B9] Desclos-Théveniau M. Coquet L. Jouenne T. Etienne P. (2015). Proteomic analysis of residual proteins in blades and petioles of fallen leaf of *Brassica napus.* *Plant Biol.* 17 408–418. 10.1111/plb.12241 25294336

[B10] Dluzniewska P. Gessler A. Kopriva S. Strnad M. Novák O. Dietrich H. (2006). Exogenous supply of glutamine and active cytokinin to the roots reduces NO3- uptake rates in poplar. *Plant Cell Environ.* 27 1284–1297. 10.1111/j.1365-3040.2006.01507.x 17080950

[B11] Eneji A. E. Inanaga S. Muranaka S. Li J. Hattori T. An P. (2008). Growth and nutrient use in four grasses under drought stress as mediated by silicon fertilizers. *J. Plant Nutr.* 31 355–365. 10.1080/01904160801894913

[B12] Epstein E. (1994). The anomaly of silicon in plant biology. *Proc. Natl. Acad. Sci. U.S.A.* 91 11–17. 11607449 10.1073/pnas.91.1.11 PMC42876

[B13] Epstein E. (1999). Silicon. *Annu. Rev. Plant Biol.* 50 641–664. 10.1146/annurev.arplant.50.1.641 15012222

[B14] Farshidi M. Abdolzadeh A. Sadeghipour H. R. (2012). Silicon nutrition alleviates physiological disorders imposed by salinity in hydroponically grown canola (*Brassica napus* L.) plants. *Acta Physiol. Planta.* 34 1779–1788.

[B15] Fauteux F. Chain F. Belzile F. Menzies J. G. Belanger R. R. (2006). The protective role of silicon in the *Arabidopsis*-powdery mildew pathosystem. *Proc. Natl. Acad. Sci. U.S.A.* 103 17554–17559. 10.1073/pnas.0606330103 17082308 PMC1859967

[B16] Fauteux F. Rémus-Borel W. Menzies J. Bélanger R. R. (2005). Silicon and plant disease resistance against pathogenic fungi. *FEMS Microbiol. Lett.* 249 1–6. 10.1016/j.femsle.2005.06.034 16006059

[B17] Fawe A. Menzies J. Chérif M. Bélanger R. R. (2001). Silicon and disease resistance in dicotyledons. *Stud. Plant Sci.* 8 159–169. 10.1016/S0928-3420(01)80013-6

[B18] Feng H. Yan M. Fan X. Li B. Shen Q. Miller A. J. (2011). Spatial expression and regulation of rice high-affinity nitrate transporters by nitrogen and carbon status. *J. Exp. Bot.* 62 2319–2332. 10.1093/jxb/erq403 21220781

[B19] Gessler A. Rennenberg H. Kopriva S. (2004). Regulation of nitrate uptake on the whole plant level: interactions between nitrogen compounds, cytokinins and carbon metabolism. *Tree Physiol.* 24 1313–1321. 10.1093/treephys/24.12.1313 15465694

[B20] Gombert J. Etienne P. Ourry A. Le Dily F. (2006). The expression patterns of SAG12/Cab genes reveal the spatial and temporal progression of leaf senescence in *Brassica napus* L. with sensitivity to the environment. *J. Exp. Bot.* 57 1949–1956. 10.1093/jxb/erj142 16720615

[B21] Gottardi S. Iacuzzo F. Tomasi N. Cortella G. Manzocco L. Pinton R. (2012). Beneficial effects of silicon on hydroponically grown corn salad (*Valerianella locusta* (L.) Laterr) plants. *Plant Physiol. Biochem.* 56 14–23. 10.1016/j.plaphy.2012.04.002 22579940

[B22] Gregersen P. L. Culetic A. Boschian L. Krupinska K. (2013). Plant senescence and crop productivity. *Plant Mol. Biol.* 82 603–622. 10.1007/s11103-013-0013-8 23354836

[B23] Guntzer F. Keller C. Meunier J. D. (2012). Benefits of plant silicon for crops: a review. *Agron. Sustain. Dev.* 32 201–213.

[B24] Habibi G. (2016). Effect of foliar-applied silicon on photochemistry, antioxidant capacity and growth in maize plant subjected to chilling stress. *Acta Agric. Slov.* 107 33–43. 10.14720/aas.2016.107.1.04

[B25] Hasanuzzaman M. Hossain M. A. Fujita M. (2011). Selenium-induced up-regulation of the antioxidant defense and methylglyoxal detoxification system reduces salinity-induced damage in rapeseed seedlings. *Biol. Trace Elem. Res.* 143 1704–1721. 10.1007/s12011-011-8958-4 21264525

[B26] Hasanuzzaman M. Nahar K. Anee T. I. Fujita M. (2017). Exogenous silicon attenuates cadmium-induced oxidative stress in *Brassica napus* L. by modeling AsA-GSH pathway and glyoxylate system. *Front. Plant Sci.* 8:1061. 10.3389/fpls.2017.01061 28674552 PMC5475239

[B27] Hashemi A. Abdolzadeh A. Sadeghipour H. R. (2010). Beneficial effects of silicon nutrition in alleviating salinity stress in hydroponically grown canola, *Brassica napus* L., plants. *Soil Sci. Plant Nutr.* 56 244–253. 10.1111/j.1747-0765.2009.00443.x

[B28] Ishiguro K. (2001). Review of research in Japan on the roles of silicon in conferring resistance against rice blast. *Stud. Plant Sci.* 8 277–291. 10.1016/S0928-3420(01)80021-5

[B29] Jones L. H. P. Handreck K. A. (1967). Silica in soils, plants, and animals. *Adv. Agron.* 19 107–149. 10.1016/S0065-2113(08)60734-8

[B30] Korndorfer G. H. Lepsch I. I. (2001). “Effect of silicon on plant growth and crop yield,” in *Silicon in Agriculture* Vol. 8 eds Datonoff L. Korndorfer G. Synder G. N. Y. (New York, NY: Elsevier Science) 17–39.

[B31] Latef A. A. A. Tran L. S. P. (2016). Impacts of priming with silicon on the growth and tolerance of maize plants to alkaline stress. *Front. Plant Sci.* 7:243. 10.3389/fpls.2016.00243 27014283 PMC4785188

[B32] Lejay L. Gansel X. Cerezo M. Tillard P. Müller C. Krapp A. (2003). Regulation of root ion transporters by photosynthesis: functional importance and relation with hexokinase. *Plant Cell* 15 2218–2232. 10.1105/tpc.013516 12953122 PMC181342

[B33] Livak K. J. Schmittgen T. D. (2001). Analysis of relative gene expression data using real-time quantitative PCR and the 2- ΔΔC_T_ method. *Methods* 25 402–408. 10.1006/meth.2001.1262 11846609

[B34] Ma J. F. Yamaji N. (2006). Silicon uptake and accumulation in higher plants. *Trends Plant Sci.* 11 392–397. 10.1016/j.tplants.2006.06.007 16839801

[B35] Ma J. F. Yamaji N. Mitani N. Tamai K. Konishi S. Fujiwara T. (2007). An efflux transporter of silicon in rice. *Nature* 448 209–212. 10.1038/nature05964 17625566

[B36] Malagoli P. Laîné P. Rossato L. Ourry A. (2005a). Dynamics of nitrogen uptake and mobilization in field-grown winter oilseed rape (*Brassica napus*) from stem extension to harvest: I. Global N flows between vegetative and reproductive tissues in relation to leaf fall and their residual N. *Ann. Bot.* 95 853–861. 10.1093/aob/mci091 15701662 PMC4246740

[B37] Malagoli P. Laîné P. Rossato L. Ourry A. (2005b). Dynamics of nitrogen uptake and mobilization in field-grown winter oilseed rape (*Brassica napus*) from stem extension to harvest. II. A 15N-labelling based simulation model of N partitioning between vegetative and reproductive tissues. *Ann. Bot.* 95 1187–1198. 10.1093/aob/mci131 15802311 PMC4246903

[B38] Markovich O. Steiner E. Kouřil S. Tarkowski P. Aharoni A. Elbaum R. (2017). Silicon promotes cytokinin biosynthesis and delays senescence in *Arabidopsis* and Sorghum. *Plant Cell Environ.* 10 1189–1196. 10.1111/pce.12913 28102542

[B39] Meyer J. H. Keeping M. G. (2001). Past, present and future research of the role of silicon for sugarcane in southern Africa. *Stud. Plant Sci.* 8 257–275. 10.1016/S0928-3420(01)80020-3

[B40] Nicot N. Hausman J. F. Hoffmann L. Evers D. (2005). Housekeeping gene selection for real-time RT-PCR normalization in potato during biotic and abiotic stress. *J. Exp. Bot.* 56 2907–2914. 10.1093/jxb/eri285 16188960

[B52] Ray S. Mondal W. A. Choudhuri M. A. (1983). Regulation of leaf senescence, grain-filling and yield of rice by kinetin and abscisic acid. *Physiol. Plant.* 59 343–346. 10.1111/j.1399-3054.1983.tb04212.x

[B41] Rodrigues F. A. Resende R. S. Dallagnol L. J. Datnoff L. E. (2015). “Silicon potentiates host defense mechanisms against infection by plant pathogens,” in *Silicon and Plant Diseases* eds Rodrigues F. A. Datnoff L. E. (Berlin: Springer) 109–138.

[B42] Rossato L. Laîné P. Ourry A. (2001). Nitrogen storage and remobilization in *Brassica napus* L. during the growth cycle: nitrogen fluxes within the plant and changes in soluble protein patterns. *J. Exp. Bot.* 52 1655–1663. 10.1093/jexbot/52.361.1655 11479330

[B43] Ruiz-Espinoza F. H. Murillo-Amador B. García-Hernández J. L. Fenech-Larios L. Rueda-Puente E. O. Troyo-Diéguez E. (2010). Field evaluation of the relationship between chlorophyll content in basil leaves and portable chlorophyll meter (SPAD-502) readings. *J. Plant Nutr.* 33 423–438. 10.1080/01904160903470463

[B44] Sakakibara H. Takei K. Hirose N. (2006). Interactions between nitrogen and cytokinin in the regulation of metabolism and development. *Trends Plants Sci.* 11 440–448. 10.1016/j.tplants.2006.07.004 16899391

[B45] Samuels A. Glass A. D. M. Ehret D. L. Menzies J. G. (1991). Mobility and deposition of silicon in cucumber plants. *Plant Cell Environ.* 14 485–492. 10.1111/j.1365-3040.1991.tb01518.x

[B46] Savant N. K. Snyder G. H. Datnoff L. E. (1997). Silicon management and sustainable rice production. *Adv. Agron.* 58 151–199. 10.1016/S0065-2113(08)60255-2

[B47] Schmidt R. E. Zhang X. Chalmeurs D. R. (1999). Response of photosynthesis and superoxide dismutase to silica apply to creeping bentgrass grown under to fertility levels. *J. Plant Nutr.* 22 1763–1773. 10.1080/01904169909365752

[B48] Tuna A. L. Kaya C. Higgs D. Murillo-Amador B. Aydemir S. Girgin A. R. (2008). Silicon improves salinity tolerance in wheat plants. *Environ. Exp. Bot.* 62 10–16. 10.1016/j.envexpbot.2007.06.006

[B49] Van Bockhaven J. Spíchal L. Novák O. Strnad M. Asano T. Kikuchi S. (2015). Silicon induces resistance to the brown spot fungus *Cochliobolus miyabeanus* by preventing the pathogen from hijacking the rice ethylene pathway. *New Phytol.* 206 761–773. 10.1111/nph.13270 25625327

[B50] Yu C. W. Murphy T. M. Lin C. H. (2003). Hydrogen peroxide-induces chilling tolerance in mung beans mediated trough ABA-independent glutathione accumulation. *Funct. Plant Biol.* 30 955–963. 10.1071/FP03091 32689080

[B51] Zimmermann P. Zentgraf U. (2005). The correlation between oxidative stress and leaf senescence during plant development. *Cell. Mol. Biol. Lett.* 10 515–534. 16217560

