# Supplementary material for: Silicon Promotes Growth of Brassica napus L. and Delays Leaf Senescence Induced by Nitrogen Starvation
Source: Front Plant Sci. 2018 Apr 23;9:516. doi: 10.3389/fpls.2018.00516 (PMC5925743; doi:10.3389/fpls.2018.00516)
Supplement: FIGURE S1 — Phenotype of Brassica napus L. plants cultivated without (-Si+N; A) or with silicon (+Si+N; B) during 1 week (D0) and grown without N for 12 days (-Si-N and +Si-N; C,D). The black arrows indicate the mature leaf identified at the beginning of the N starvation (D0) and used to follow the time of course of photosynthetic activity, SPAD measurements senescence progression (BnaSAG12/Cab). [file Image_1.PDF]

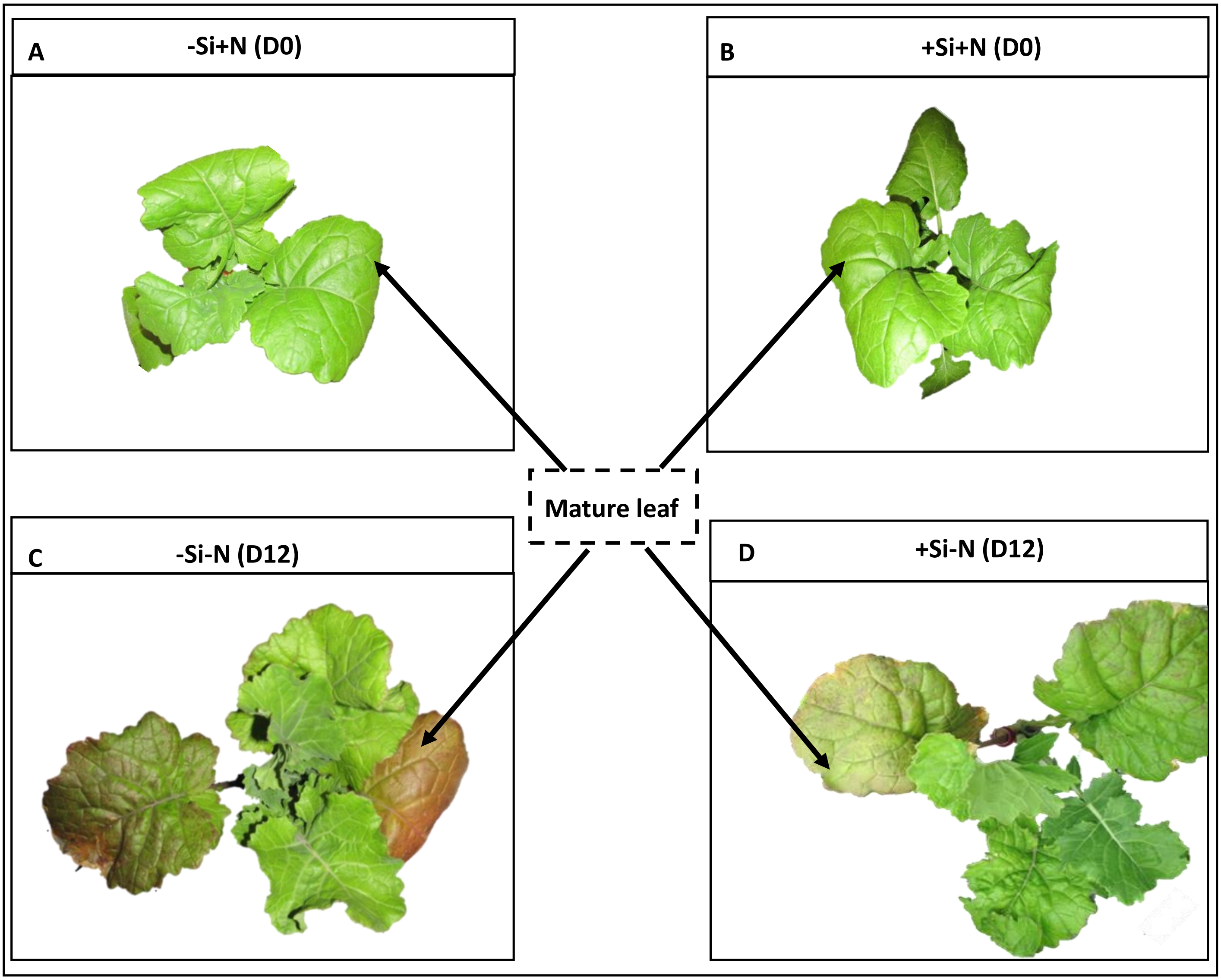

Supplementary Figure S1: Phenotype of *Brassica napus.* L plants cultivated without (-Si+N; A) or with silicon (+Si+N; B) during one week (D0) and grown without N for 12 days (-Si-N and +Si-N; C and D). The black arrows indicate the mature leaf identified at the beginning of the N starvation (D0) and used to follow the time of course of photosynthetic activity, SPAD measurements senescence progression (*BnaSAG12*/*Cab*).

Haddad et al., Supplementary figure S1
