# Supplementary figures and images for: Silicon Promotes Growth of Brassica napus L. and Delays Leaf Senescence Induced by Nitrogen Starvation
Source: Front Plant Sci. 2018 Apr 23;9:516. doi: 10.3389/fpls.2018.00516 (PMC5925743; doi:10.3389/fpls.2018.00516)

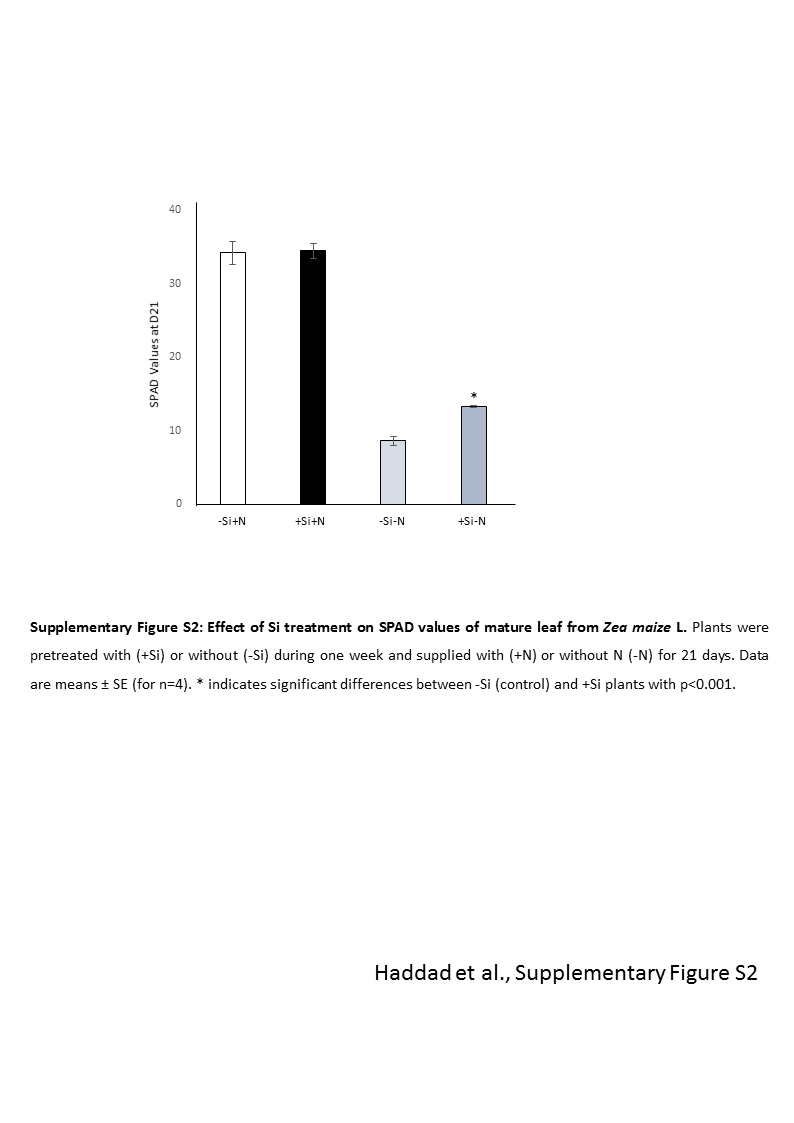

Supplement: FIGURE S2 — Effect of Si treatment on SPAD values of mature leaf from Zea mays L. Plants were pretreated with (+Si) or without (-Si) during 1 week and supplied with (+N) or without N (-N) for 21 days (D21). Data are means ± SE (for n = 4). ∗ indicates significant differences between -Si (control) and +Si plants with p < 0.001. [file Image_2.TIF]

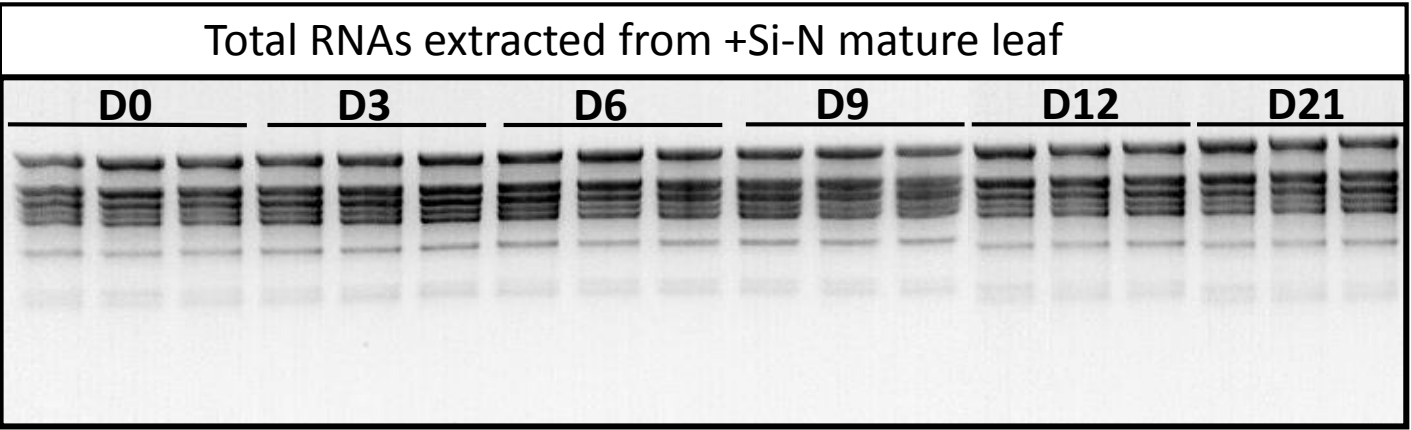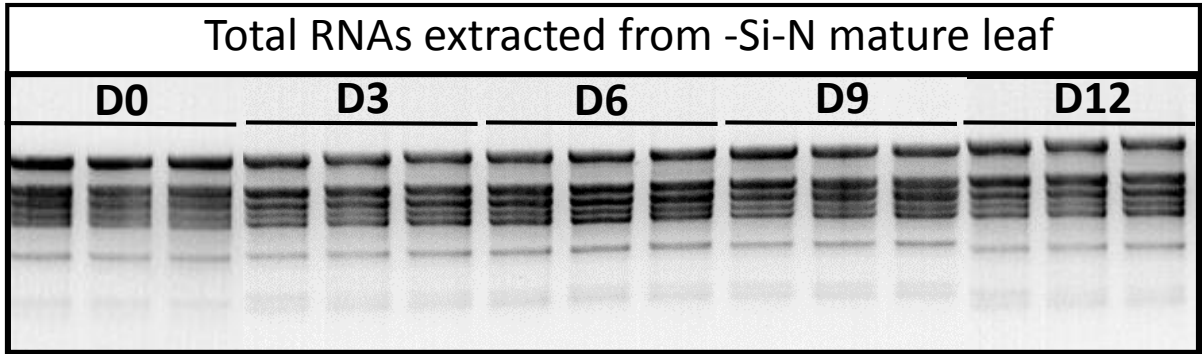

Supplement: FIGURE S3 — Control quality of total RNAs extracted from mature leaf of N (-N) deprived plants treated with (+Si) or without (-Si). At each time (Day 0, 3, 6, 9, 12, and 21 only for +Si-N), three biological repetitions have been performed. 1 μg of total RNAs were separated on agarose gel (1%) containing 0.5 μg/ml of ethidium bromide (0.5 μg/ml). After separation, agarose gels were scanned under UV light with a Gel DocTM EZ scanner (Bio-Rad, Marnes-la-Coquette, France) and visualized using ImageLabTM software (Bio-Rad, Marnes-la-Coquette, France). [file Image_3.PDF]
