# Supplementary material for: Silicon Promotes Growth of Brassica napus L. and Delays Leaf Senescence Induced by Nitrogen Starvation
Source: Front Plant Sci. 2018 Apr 23;9:516. doi: 10.3389/fpls.2018.00516 (PMC5925743; doi:10.3389/fpls.2018.00516)
Supplement: TABLE S1 — Detail of cytokinin contents [expressed in pg mg-1 dry weight (DW)] in roots and shoots (D0) and in mature leaf and roots (D12) of Brassica napus L. Plants were pretreated with (+Si) or without (-Si) silicon for 1 week and supplied with (-Si+N; +Si+N) or without (-Si-N; +Si-N) N for 12 days (from D0 to D12). Data are means ± SE (for n = 3). ∗ and ∗∗ indicate significant differences (in bold) between -Si and +Si plants with p < 0.01 and p < 0.001, respectively. IP, N6-Isopentenyladenine; t-Z, Trans-zeatin; IPAdo, N6-Isopentenyladenosine. [file Table_1.PDF]

Supplementary Table S1: Detail of cytokinin contents (expressed in pg mg^{-1} DW) in roots and shoots (D0) and in mature leaf and roots (D12) of *Brassica napus* L plants pretreated with (+Si) or without (-Si) silicon for one week and supplied with (-Si+N; +Si+N) or without (-Si-N; +Si-N) nitrogen for 12 days (from D0 to D12). Data are means ± SE (for n=3). * and ** indicate significant differences (in bold) between -Si and +Si plants with p<0.01 and p<0.001, respectively. IP: N_6-Isopentenyladenine; t-Z: trans-zeatin; IPAdo: N_6-Isopentenyladenosine.

Harvest time	Organ	Treatment	IP (pg mg^{-1} DW)	t-Z (pg mg^{-1} DW	IPA do (pg mg^{-1} DW)
D0	Shoots	-Si (Control)	0.015 ±0.002	0.010 ±0.002	0.025 ±0.001
		+Si	0.012 ±0.001	0.007 ±0.001	0.021 ±0.002
	Roots	-Si (Control)	0.097 ±0.001	**0.043 ±0.002***	0.369 ±0.009
		+Si	0.102 ±0.001	**0.020 ±0.001**	0.328 ±0.011
D12	Mature leaf	-Si+N	0.028 ±0.001*	0.059 ±0.016	0.046 ±0.006
		+Si+N	0.018 ±0.001	0.017 ±0.000	0.033 ±0.000
		-Si-N	**0.012 ±0.001****	0.055 ±0.024	0.199 ±0.104
		+Si-N	**0.022 ±0.001**	0.040 ±0.018	0.225 ±0.176
	Roots	-Si+N	0.095 ±0.001	0.012 ±0.001	**0.303 ±0.013****
		+Si+N	0.090 ±0.002	0.014 ±0.001	**0.414 ±0.024**
		-Si-N	0.056 ±0.002	0.007 ±0.001	0.101 ±0.001
		+Si-N	0.042 ±0.001	0.006 ±0.000	0.059 ±0.003
